# Density‐weighted concentric rings *k*‐space trajectory for ^1^H magnetic resonance spectroscopic imaging at 7 T

**DOI:** 10.1002/nbm.3838

**Published:** 2017-10-18

**Authors:** Mark Chiew, Wenwen Jiang, Brian Burns, Peder Larson, Adam Steel, Peter Jezzard, M. Albert Thomas, Uzay E. Emir

**Affiliations:** ^1^ Wellcome Centre for Integrative Neuroimaging, FMRIB Division, Nuffield Department of Clinical Neurosciences University of Oxford John Radcliffe Hospital Oxford UK; ^2^ Department of Radiology and Biomedical Imaging University of California San Francisco CA USA; ^3^ Department of Oncology University of Oxford Oxford UK; ^4^ Laboratory of Brain and Cognition, National Institute of Mental Health National Institutes of Health Bethesda MD USA; ^5^ Department of Radiological Sciences University of California Los Angeles CA USA; ^6^ School of Health Sciences Purdue University West Lafayette IN USA

**Keywords:** echo‐planar, spectroscopic imaging, concentric rings, spectroscopic imaging, ultra‐high field

## Abstract

It has been shown that density‐weighted (DW) k‐space sampling with spiral and conventional phase encoding trajectories reduces spatial side lobes in magnetic resonance spectroscopic imaging (MRSI). In this study, we propose a new concentric ring trajectory (CRT) for DW‐MRSI that samples k‐space with a density that is proportional to a spatial, isotropic Hanning window. The properties of two different DW‐CRTs were compared against a radially equidistant (RE) CRT and an echo‐planar spectroscopic imaging (EPSI) trajectory in simulations, phantoms and in vivo experiments. These experiments, conducted at 7 T with a fixed nominal voxel size and matched acquisition times, revealed that the two DW‐CRT designs improved the shape of the spatial response function by suppressing side lobes, also resulting in improved signal‐to‐noise ratio (SNR). High‐quality spectra were acquired for all trajectories from a specific region of interest in the motor cortex with an in‐plane resolution of 7.5 × 7.5 mm^2^ in 8 min 3 s. Due to hardware limitations, high‐spatial‐resolution spectra with an in‐plane resolution of 5 × 5 mm^2^ and an acquisition time of 12 min 48 s were acquired only for the RE and one of the DW‐CRT trajectories and not for EPSI. For all phantom and in vivo experiments, DW‐CRTs resulted in the highest SNR. The achieved in vivo spectral quality of the DW‐CRT method allowed for reliable metabolic mapping of eight metabolites including N‐acetylaspartylglutamate, γ‐aminobutyric acid and glutathione with Cramér‐Rao lower bounds below 50%, using an LCModel analysis. Finally, high‐quality metabolic mapping of a whole brain slice using DW‐CRT was achieved with a high in‐plane resolution of 5 × 5 mm^2^ in a healthy subject. These findings demonstrate that our DW‐CRT MRSI technique can perform robustly on MRI systems and within a clinically feasible acquisition time.

**Abbreviations used**
2D: two dimensional
ANOVA: analysis of variance
CRLB: Cramér‐Rao lower bound
CRT: concentric ring trajectory
DW: density weighted
EPSI: echo‐planar spectroscopic imaging
FID: free induction decay
FOV: field of view
FWHM: full width at half maximum
GABA: γ‐aminobutyric acid
Gln: glutamine
Glu: glutamate
GM: gray matter
GPC: glycerophosphocholine
GRESHIM: gradient‐echo shimming
GSH: glutathione
HLSVD: Hankel‐Lanczos singular value decomposition
Lac: lactate
MRSI: magnetic resonance spectroscopic imaging
*myo*‐Ins: *myo*‐inositol
NAA: N‐acetylaspartate
NAAG: N‐acetylaspartylglutamate
OVS: outer volume suppression
PCho: phosphocholine
PCr: phosphocreatine
PSF: point spread function
RE: radially equidistant
SAR: specific absorption rate
SBW: spectral bandwidth
SD: standard deviation
SE: spin‐echo
SEM: standard error of the mean
semi‐LASER: semi‐localization by adiabatic selective refocusing
SNR: signal‐to‐noise ratio
SRF: spatial response function
tCho: total choline
tCr: total creatine
UHF: ultra‐high field
VOI: volume of interest
WM: white matter

## 1 INTRODUCTION

Complementary to MRI, non‐invasive MRS techniques have been valuable in revealing abnormalities before any visible macroscopic changes in brain anatomy, physiology or function occur in neurological disorders, as they provide unique information on the neurochemical composition of brain tissue. Magnetic resonance spectroscopic imaging (MRSI) offers the advantage of non‐invasively collecting neurochemical profiles over larger regions than single‐voxel MRS, and at higher spatial resolutions when the disease is not focal and the region of interest is not known.^1^ However, the practicality of MRSI methods is compromised by low concentrations of metabolites relative to water, leading to low signal‐to‐noise ratios (SNRs) as well as long acquisition times due to spectral and spatial encoding. At ultra‐high fields (UHFs, ≥7 T), MRSI benefits from increased SNR, and the possibility of quantifying more metabolites as a result of better resolved neighboring resonances. These gains could translate to higher‐spatial‐resolution MRSI.^2^

There have been several methods proposed to accelerate acquisition duration for MRSI at UHF. One of the first acceleration methods for *in vivo* MRSI at 7 T was two‐dimensional (2D) spin‐echo (SE) echo‐planar spectroscopic imaging (EPSI), which has been followed by fast phase encoding trajectories using non‐echo‐planar (spiral,^3^ rosette^4^ and concentric ring trajectory (CRT)^5^ spectroscopic imaging), all proposed to reduce acquisition time. Another method for reducing the acquisition duration of MRSI is to decrease the specific absorption rate (SAR) of the sequence to achieve short repetition times (*T*_R_) < 1000 ms, which has been demonstrated for free induction decay (FID) ^1^H MRSI at 7 T,^6^, ^7^ and 9.4 T.^8^ For instance, metabolite maps at 9.4 T have been successfully obtained at both high (voxel size of 97.6 μL) and ultra‐high (voxel size of 24.4 μL) spatial resolutions in scan times of 11 and 46 min, respectively.^8^

In addition to long acquisition times and low SNR, MRSI methods can suffer from significant side lobe artifacts in their spatial response functions (SRFs). These artifacts lead to contamination of undesired signals from the subcutaneous lipid layer and voxel bleeding, which degrade the desired metabolite spectra. To suppress lipid signals, the most common methods are outer volume suppression (OVS),^9^ inversion recovery^10^ and selective brain‐only excitation.^11^ Another method is removing the lipid signal via post‐processing.^12^, ^13^ An alternative approach to reduce spatial contamination is to acquire MRSI data with a density‐weighted (DW) pattern^14^, ^15^ to shape the SRF, given by the Fourier transform of the sampling density pattern. Initially, DW methods were implemented for MRSI with repeated phase encodings, which resulted in very long acquisition times.^16^ However, significant reductions in acquisition time and SRF side lobe contamination were achieved when DW *k*‐space sampling with spiral‐based trajectories was used for MRSI.^15^

It has been shown that the use of a CRT for MRSI is more time efficient compared with EPSI, and less sensitive to system imperfections compared with spiral MRSI.^17^, ^18^ In addition, the CRT approach is well suited to implementing DW sampling with an isotropic SRF, because these patterns are rotationally symmetric in *k*‐space. The intent of this work, therefore, is to develop a DW MRSI acquisition technique at 7 T that addresses the challenges of speed, spatial resolution and side lobe artifacts. Thus, we propose to use CRT‐based MRSI to sample *k*‐space with a density proportional to an isotropic, spatial Hanning window.^19^ In this work, we also demonstrate the implementation of DW‐CRT at 7 T through a quantitative comparison with radially equidistant (RE) CRT and EPSI acquisitions.

## 2 METHODS

### 2.1 *k*‐space trajectory designs for MRSI

#### 2.1.1 EPSI

**Figure 1:**
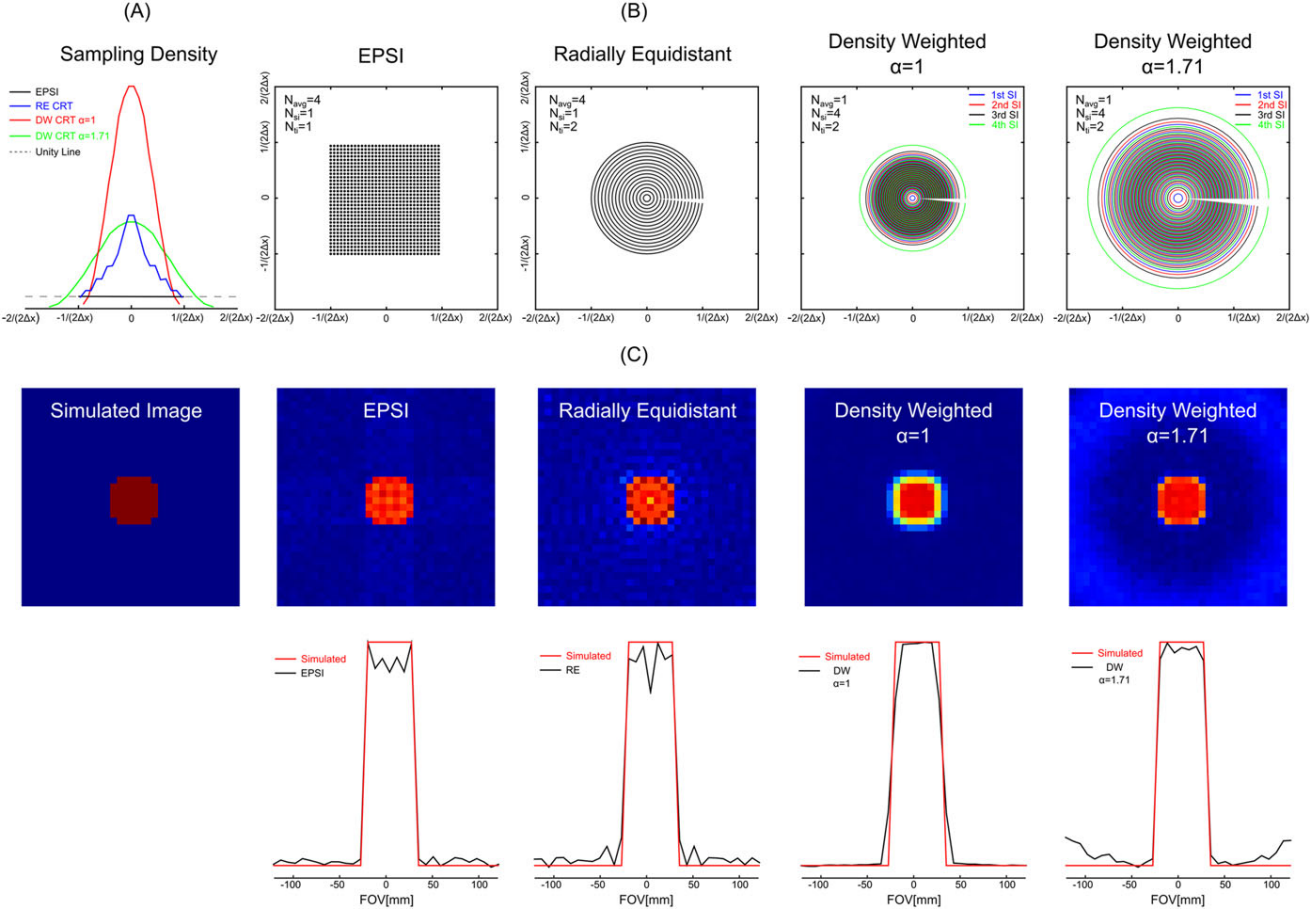
A, sampling densities for EPSI, RE and DW trajectories. B, *k*‐space trajectories of EPSI, RE and DW concentric ring MRSI for 32 × 32 pixel extent in (*k_x_*, *k_y_*) space of over 240 mm × 240 mm FOV. C, simulation comparison of different *k*‐space trajectories' 2D SRFs (top) and 1D SRF profiles along the *x*‐axis (bottom). Simulations were performed by reconstructing a constant object with a Gaussian noise at the origin in a grid of 32 × 32 (FOV = 240 mm × 240 mm with a nominal voxel dimension of 0.56 mL) using NUFFT

The echo‐planar *k*‐space trajectory for MRSI (EPSI) (Figure 1A) was generated with phase encoding along one spatial dimension (*k_y_*) and an oscillating readout gradient along the other (*k_x_*‐*t* plane).^20^ The EPSI *k*‐space data are scanned in a zigzag *k_x_*‐*t* trajectory and collected using both odd and even echoes.

#### 2.1.2 RE CRT

A RE‐CRT for MRSI was derived similarly to Furuyama et al.^18^ The following equations were used to generate the gradient waveforms along the *x* (*G_x_*) and *y* (*G_y_*) directions:

(1) $$\begin{array}{l} G_{x}\left(t\right)=-\frac{4\pi^{2}k_{n}}{\mathrm{\gamma T}}\times \cos\left[\frac{2\pi }{T}\left(t-T_{E}\right)\right]\\ G_{y}\left(t\right)=-\frac{4\pi^{2}k_{n}}{\mathrm{\gamma T}}\times \sin\left[\frac{2\pi }{T}\left(t-T_{E}\right)\right] \end{array}$$

where *T* represents the duration of the circle, and *k_n_* = (*n* + 1/2)/FOV represents the radius of the circle with *n* = 0, 1, 2, …, *n*_max_ (Figure 1A).

#### 2.1.3 DW CRT

The DW acquisition method was introduced based on altering the density of sampled points in *k*‐space.^15^ A Hanning‐window density weighting was used to determine the radius of each ring, since it has been shown to provide a good compromise between sensitivity and spatial resolution.^19^ The function used is given by

(2) $$w\left(k\right)=\frac{\Delta x}{2}\left(1+\cos\left(2\mathrm{\pi k}\Delta x/\alpha \right)\;\right)$$

where Δ*x* is the nominal spatial resolution. To achieve the desired density, *w*(*k*) is integrated from *k* = 0 and the *k* locations corresponding to equal area bins are taken as the radii for the sampling rings. In this equation, the parameter *α* controls the adjustment of the effective spatial resolution by modulating the width of the SRF main lobe, or equivalently the extent of *k*‐space sampling. Depending on the value of *α* (*α* > 1), the DW‐CRT spans a greater extent in *k*‐space (−*α*/(2Δ*x*) < *k_n_* < *α*/(2Δ*x*)) because it samples the outer *k*‐space region coarsely (Figure 1A). However, when *α* is set to 1, DW‐CRTs are distributed between −1/(2Δ*x*) and 1/(2Δ*x*).

### 2.2 Simulations

To assess the performance of DW‐CRT against the other trajectories, EPSI and RE‐CRT, simulations were performed to assess the level of side lobes and SNR performance of each approach. A simple, constant object with added Gaussian noise was positioned at the origin of a 32 × 32 grid (field of view, FOV = 240 mm × 240 mm with a nominal voxel dimension of 7.5 mm) and reconstructed using the non‐uniform FFT (NUFFT) toolbox with min‐max Kaiser‐Bessel kernel interpolation and twofold oversampling.^21^ In order to keep the simulation parameters identical to *in vivo* acquisition parameters (Table 1), four averages (number of averages, *N*_avg_ = 4) of 16 rings (number of rings, *N*_ring_ = 16) were used for RE‐CRT reconstruction (16 unique radii), whereas DW‐CRT simulations were performed using 16 rings and four spatial interleaves (64 unique radii, number of spatial interleaves, *N*_si_ = 4) with two different *α* values, 1 and 1.71, as shown in Equation (2). The *α* value of 1.71 was calculated by defining the nominal spatial resolution as the 64% width of the SRF,^22^ whereas the *α* value of 1 was chosen to alleviate potential constraints on the gradient slew rate during the high‐resolution MRSI experiments. For EPSI, a 32 × 32 uniform Cartesian grid was used for reconstruction with NUFFT gridding.

**Table 1:** List of all MRSI trajectories and their specifications. *N*_avg_, number of averages; *N*_ti_, number of temporal interleaves; *N*_si_, number of spatial interleaves; *N*_ring_, number of rings; *N_y_*, number of phase encoding steps; NA, not applicable, *T*_acq_, total acquisition time

	2D matrix	*N*_avg_	*N*_ti_	*N*_si_	*N*_ring_	N_y_	SBW (Hz)	*T*_acq_ (min:s)
EPSI	32 × 32	4	1	1	NA	32	2380	8:32
Re‐CRT	32 × 32	4	2	1	16	NA	2500	8:32
DW‐CRT with *α* = 1	32 × 32	1	2	4	16	NA	2500	8:32
DW‐CRT with *α* = 1.71	32 × 32	1	2	4	16	NA	2500	8:32
Re‐CRT	48 × 48	4	2	1	24	NA	2500	12:48
DW‐CRT with *α* = 1	48 × 48	1	2	4	24	NA	2500	12:48

In this study, the SRF is used rather than the point spread function (PSF) since it provides information about side lobes and the signal contamination between adjacent voxels, whereas the PSF describes how signal from a single source point in the object contributes to all voxels of the entire image.

### 2.3 Experimental comparison

Phantom and *in vivo* studies were conducted on a 7 T whole body MR system (Siemens, Erlangen, Germany) with a Nova Medical 32‐channel receive array (*N*_cha_) head‐coil, maximum gradient 70 mT/m, and maximum slew rate of 200 mT/m/ms. Five healthy volunteers (three males/two females, aged 28.8 ± 3.4 (mean ± standard deviation, SD, years), participated in this study after giving informed consent under an institutionally approved technical development protocol.

### 2.4 *In vivo* MRSI acquisitions

All *in vivo* 2D MRSI scans from a specific region of interest in the motor cortex were positioned manually using a high‐resolution *T*_1_‐weighted MP‐RAGE image (repetition time *T*_R_ = 2.3 s, inversion time *T*_I_ = 1.05 s, echo time *T*_E_ = 2.8 ms, total acquisition time = 3 min). Spectra were measured with a semi‐localization by adiabatic selective refocusing (semi‐LASER) pulse sequence (*T*_E_ = 36 ms, *T*_R_ = 4 s) with VAPOR (variable power and optimized relaxation delays) water suppression and OVS.^23^ Localization was performed by an asymmetric slice‐selective excitation pulse (duration = 6 ms) and four slice‐selective adiabatic‐full‐passage refocusing pulses (6 ms duration, HS4 modulation, R25)^24^ as performed previously.^25^, ^26^ The semi‐LASER localization was used to excite a 110 mm × 80 mm × 10 mm region centrally within the FOV. The imaging box was localized with a FOV of 240 mm × 240 mm and a slice thickness of 10 mm. First‐ and second‐order shims were first adjusted by gradient‐echo shimming (GRESHIM).^27^ GRESHIM acquires a *B*_0_ field map using gradient‐echo images with two different echo times in order to calculate the required shim currents.^27^ A second step involved only fine adjustment of first‐order shims using FASTMAP.^28^

### 2.5 EPSI

For the EPSI trajectory, 32 phase‐encoded (number of phase encoding steps, *N_y_* = 32) excitations were required to cover a FOV of 240 mm × 240 mm. To cover a 32 × 32 grid, 32 points per readout (number of readout points, *N_x_* = 32) resulting in an individual voxel of 0.56 mL were collected with an ADC bandwidth of 100 kHz, a ramp time of 100 μs and a maximum slew rate of 195.7 mT/m/ms. Using 512 even and 512 odd echoes, a total of 1024 spectral points (number of spectral points, *N*_sp_ = 1024) were collected for an effective spectral bandwidth (SBW) of 2380 Hz (Supporting Figure 1a). The total acquisition time was completed with four averages (*N*_avg_ = 4) in 8 min 32 s (*N_y_N*_avg_ *T*_R_ = 512 s). Only a fully excited 13 × 9 voxel region was used for quantitative analysis.

### 2.6 CRT

For all CRT designs, 64 points per ring (number of points per ring, *N*_p_ring_ = 64) were collected with an ADC bandwidth of 80 kHz.^29^ 1024 spectral points (*N*_sp_ = 1024) with two temporal interleaves (number of temporal interleaves, *N*_ti_ = 2) were collected with an SBW of 2500 Hz. Temporal interleaves were implemented by inverting the readout trajectory (Supporting Figure 1b).

To cover the 32 × 32 grid, 16 RE concentric rings (*N*_ring_ = 16), resulting in a nominal individual voxel size of 0.56 mL, were acquired with four averages (*N*_avg_ = 4) in 8 min 32 s (*N*_ring_ *N*_avg_ *N*_ti_ *T*_R_ = 512 s, maximum slew rate = 92 mT/m/ms). As for DW‐CRT, four spatial interleaves (*N*_si_ = 4 and *N*_avg_ = 1) of 16 DW concentric rings (*N*_ring_ = 16) were acquired with two different *α* values, *α* = 1 (maximum slew rate = 91.8 mT/m/ms) and *α* = 1.71 (maximum slew rate = 157 mT/m/ms). The total acquisition for DW‐CRT with four spatial interleaves was completed in 8 min 32 s (*N*_ring_ *N*_si_ *N*_avg_ *N*_ti_ *T*_R_ = 512 s), which is the same for all the trajectories that were compared, but in this case 4 × 16 64 unique radii were sampled, compared with the four averages of 16 radii in the RE trajectory. Only a fully excited 13 × 9 voxel region was used for quantitative analysis.

### 2.7 High‐resolution MRSI acquisition

High‐resolution MRSI measurements were conducted only for RE and DW‐CRT with an *α* value of 1, since DW‐CRT with *α* = 1.71 and EPSI exceeded the maximum gradient slew rate of the system for outer *k*‐space regions and fast alternating gradient of even‐odd echoes, respectively. To cover the 48 × 48 grid, 24 RE concentric rings (*N*_ring_ = 24) resulting in an individual voxel size of 0.5 mL were acquired with four averages (*N*_avg_ = 4) in 12 min and 48 s (*N*_ring_ *N*_avg_ *N*_ti_ *T*_R_ = 12 min 48 s, maximum slew rate = 140.3 mT/m/ms). For DW‐CRT, four spatially interleaved (*N*_si_ = 4 and *N*_avg_ = 1) 24 DW concentric rings were acquired with an *α* value of 1 (maximum slew rate = 138.7 mT/m/ms). The total acquisition time for DW‐CRT with four spatial interleaves was 12 min 48 s (*N*_ring_ *N*_si_ *N*_avg_ *N*_ti_ *T*_R_ = 768 s). Only a fully excited 22 × 14 voxel region was used for quantitative analysis. Although this *k*‐space sampling scheme starts to violate the azimuthal sampling criterion for polar *k*‐space sampling at the 20th ring,^30^ there is no significant loss in qualitative image quality, SNR or resolution.^29^

### 2.8 Feasibility of the whole brain slice MRSI

The feasibility of the whole brain slice with all trajectories was tested on a healthy volunteer with grids of 32 × 32 and 48 × 48 (FOV = 240 mm × 240 mm, semi‐LASER localization = 145 mm × 120 mm × 10 mm, *T*_R_ = 4 s, *T*_E_ = 36 ms). High‐resolution whole brain slice MRSI measurements with a grid of 48 × 48 were conducted only for RE and DW‐CRT with an *α* value of 1, due to maximum slew rate limitations of the hardware.

Lipid contamination was removed during post‐processing using a lipid‐basis penalty algorithm.^13^, ^31^ Briefly, an iterative lipid‐basis reconstruction with L2‐penalty was applied to the metabolite image by assuming that the metabolite spectra from the brain, and the lipid spectra from the lipid masks, were orthogonal. Unsuppressed water whole brain slice MRSI data were used to generate a lipid mask, i.e. of skull + subcutaneous fat, and a brain mask.

### 2.9 Post‐processing

All the reconstruction algorithms were implemented in MATLAB (MathWorks, Natick, MA, USA). Gridding and FFT steps were done using NUFFT as described for the simulations. In this work, we purposely designed the DW‐CRT trajectory to have a Hanning‐windowed radial sampling density by carefully selecting the location of the sampled *k*‐space radii. Doing so means that no *k*‐space density compensation^32^ is required, because the acquired density already matches the desired Hanning‐windowed density. This results in an SRF with lower peak side lobes than a density‐corrected *k*‐space, and better SNR than a post hoc smoothing or *k*‐space weighting would provide. Therefore, while density compensation was applied to the non‐Cartesian RE‐CRT *k*‐space data, NUFFT gridding was performed without using any post hoc density compensation for the DW‐CRT data, as it is already weighted by design.

To achieve the full SBW while using even and odd echoes for EPSI and inverted readout gradients for CRT, an interlaced Fourier transform was used.^33^ First, a NUFFT operator was initiated with exact *k*‐space locations and their corresponding frequencies (3D *k*‐space data *k_x_*, *k_y_* and *k*_f_). Specifically, *k*‐space locations (*k_x_*) of odd echoes and those of the even echoes were first calculated separately and subsequently concatenated to form a doubled SBW (Supporting Figure 1a). Then, NUFFT calculated the Fourier transform of the acquired 3D *k*‐space MRSI data (*k_x_*, *k_y_* and *t*) on the EPSI trajectory for all coil channels and time frames. As for CRT, the NUFFT operator was initialized with exact *k*‐space locations of the inverted and non‐inverted readout gradient trajectories, calculated separately and subsequently concatenated to form a doubled SBW (Supporting Figure 1b). Then, the NUFFT calculated the Fourier transform of the acquired 3D *k*‐space MRSI data (*k_x_*, *k_y_* and *t*) from the CRT trajectory for all coil channels and time frames. The final matrix size of the reconstructed MRSI image after NUFFT was 2*N*_ring_ × 2*N*_ring_ × *N*_sp_ × *N*_cha_ × *N*_avg_ for CRT and *N_x_* × *N_y_* × *N*_sp_ × *N*_cha_ × *N*_avg_ for EPSI.

### 2.10 Metabolite quantification

The reconstructed metabolite spectrum for each MRSI voxel was quantified using the LCModel package.^34^ For absolute quantification, eddy current and zero‐order phase correction purposes, a water reference scan using the same trajectory was acquired from each subject. The residual water peak was filtered with the Hankel‐Lanczos singular value decomposition (HLSVD) algorithm prior to the LCModel analysis.^35^ Concentrations were calculated using the unsuppressed water spectrum as an internal reference for *in vivo* data. The model spectra of alanine (Ala), aspartate (Asp), ascorbate/vitamin C (Asc), glycerophosphocholine (GPC), phosphocholine (PCho), creatine (Cr), phosphocreatine (PCr), γ‐aminobutyric acid (GABA), glucose, glutamine (Gln), glutamate (Glu), glutathione (GSH), lactate (Lac), *myo*‐inositol (*myo*‐Ins), N‐acetylaspartate (NAA), N‐acetylaspartylglutamate (NAAG), phosphoethanolamine, *scyllo*‐inositol and taurine were generated based on previously reported chemical shifts and coupling constants by the GAMMA/PyGAMMA simulation library of VeSPA (Versatile Simulation, Pulses and Analysis) according to a density matrix formalism.^36^ Simulations were performed using the same RF pulses and sequence timings as on the 7 T system in use. Eight LCModel‐simulated macromolecule resonances were included in the analysis at the following positions: 0.91, 1.21, 1.43, 1.67, 1.95, 2.08, 2.25 and 3 ppm. Concentrations were not corrected for *T*_1_ and *T*_2_ effects or cerebrospinal fluid contribution. If the correlation between two metabolites was consistently high (correlation coefficient < −0.5) in a given region, their sum was reported, e.g. total creatine (Cr + PCr, tCr) and total choline (GPC + PCho, tCho).

### 2.11 Phantom measurements

The MRSI trajectories were tested on three phantoms using the same acquisition parameters as used for *in vivo* measurements. First, the trajectories and their reconstructions were tested on a cylindrical resolution phantom (General Electric Medical Systems, Milwaukee, WI, USA). Second, a phantom measurement to assess the SRF was performed on a small water containing cylinder phantom with diameter 45 mm. Finally, a phantom experiment was performed using the larger volume of interest (VOI) of the semi‐LASER localization (120 mm × 120 mm) on an MRS ‘Braino’ phantom (General Electric Medical Systems) containing 10 mmol creatine, 3 mmol choline, 5 mmol Lac, 1 mL/L Gd‐DPTA (Magnevist), 12.5 mmol Glu, 7.5 mmol *myo*‐Ins, 12.5 mmol NAA, 0.1% sodium azide, 56 mmol sodium hydroxide and 50 mmol potassium phosphate monobasic. Only a fully excited 15 × 15 voxel region was used for quantitative SNR analysis.

### 2.12 Statistical analysis

To compare the *in vivo* performance of DW‐CRT against RE and EPSI, the SNR (SNR_lcmodel_) and linewidth (LW_lcmodel_) estimations of LCModel were used. SNR_lcmodel_ was calculated using the peak height of the NAA singlet peak and the root‐mean‐square of its residual, and LW_lcmodel_ was calculated from the width at half‐height of the singlet resonances of NAA, Cr and Cho. One‐way analysis of variance (ANOVA) with a Tukey post hoc test was consecutively applied for multiple‐comparison statistical analysis, considering *p* < 0.05 to be statistically significant. *η* ^2^ (eta squared) was used for the effect size of ANOVA. Statistical analysis was performed on those brain metabolites that had Cramér‐Rao lower bound (CRLB) goodness of fit values smaller than 50% (total N‐acetylaspartate, tCr, tCho, *myo*‐Ins and Glu + Gln). For the *in vitro* comparison using the Braino phantom, only SNR_freq_, defined as the peak height relative to RMS noise calculated in the spectral range between 5.5 and 6 ppm, was used for statistical analysis.

## 3 RESULTS

### 3.1 Simulation comparison of the DW‐CRT with EPSI and RE‐CRT

Image quality resulting from different sampling schemes can be understood through the SRF, as shown in Figure 1. The two DW‐CRTs compared with RE‐CRT and EPSI and their sampling densities are illustrated in Figure 1B,A. For the CRTs, the sampling densities were normalized to 1 at the edges of (*k_x_*, *k_y_*) space, where the spacing between adjacent data points is such that the Nyquist sampling criterion in the radial direction is satisfied exactly for the given spatial FOV.^15^, ^30^ 2D simulated SRFs and the corresponding 1D SRF profiles are also shown in Figure 1C. Although the SRFs result in approximately the same full width at half maximum (FWHM) values for all trajectories, the differences were mainly observed in the level of side lobes and the shapes of the SRFs. The resulting SRFs clearly demonstrate the reduced side lobes of the DW‐CRTs. The DW‐CRT with *α* = 1.71 and EPSI produced SRFs similar to the simulated object (Figure 1C).

When SRFs were compared between DW‐CRTs with different *α* values, the DW CRT with *α* = 1 had a 133 mm FOV near the edge of *k*‐space, whereas for *α* = 1.71 the DW CRT has a 78 mm FOV near the edge of *k*‐space. The aliasing‐free region (effective FOV) in Figure 1C for the DW CRT with *α* = 1.71 was smaller than that of DW CRT with *α* = 1, because the sampling density of the trajectory (Figure 1B) was less than unity in the outer *k*‐space region. However, with multiple spatial interleaves (taking the place of multiple averages), the increased sampling density could typically accommodate conventional FOVs. In addition, since *α* = 1 sampled fewer high‐spatial‐frequency components, it resulted in an improved SNR image (at lower effective resolution) if the object contained mostly low‐spatial‐frequency components.

### 3.2 Phantom comparison of the DW CRTs with EPSI and RE CRT

**Figure 2:**
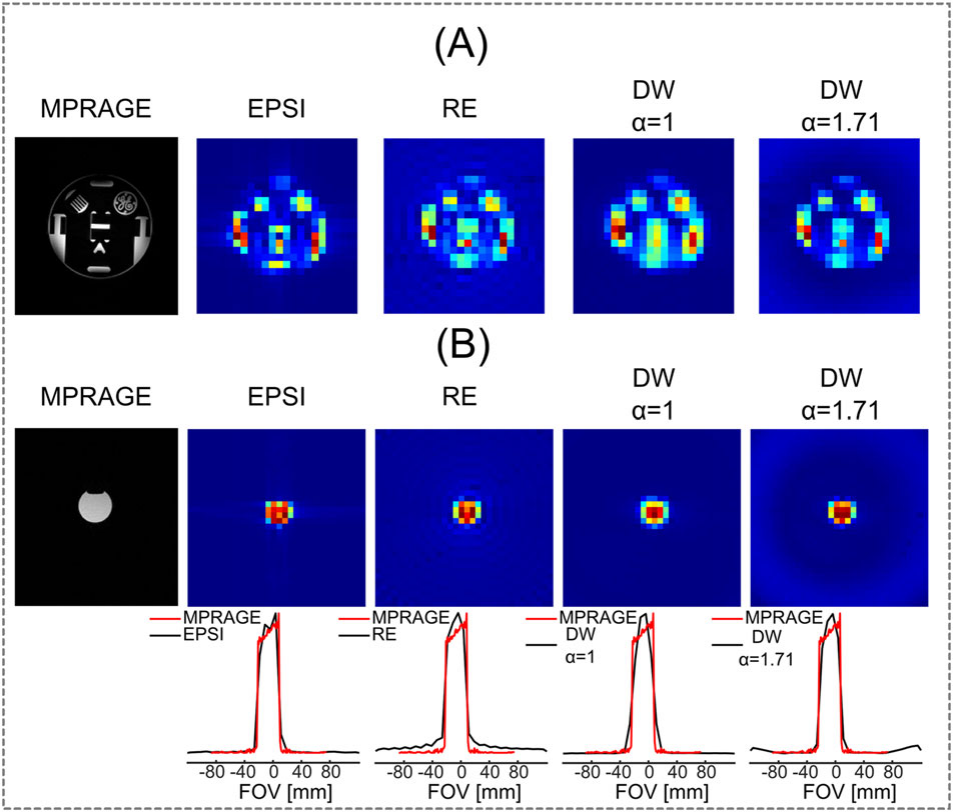
Results of resolution and SRF using phantom experiments. A, high‐resolution *T*1‐weighted MPRAGE image of the slice studied and corresponding water images of each *k*‐space trajectory with a final grid of 32 × 32 (FOV = 240 mm × 240 mm with a nominal voxel dimension of 0.56 mL) obtained using the first time point of the water FID. B, comparison of different *k*‐space trajectories' 2D SRFs (top) and 1D SRF profiles along the *x*‐axis (bottom) using the phantom

Figure 2A shows the results from the resolution phantom for the non‐water‐suppressed MRSI measurements using different trajectories. Although the final resolution of the image generated from the first time point of the FID was poorer than that of the MPRAGE image (0.56 mL versus 0.001 mL), the MRSI images using different trajectories and their reconstructions generated images with structural information similar to that of MPRAGE.

The small cylinder phantom measurements (Figure 2B) resulted in SRFs that were in agreement with simulation findings (Figure 1C). The SRFs resulted in approximately the same FWHM values, whereas their differences were mainly in the side lobes and shapes of the SRFs (Figure 2B). As with the simulations, the resulting SRFs clearly demonstrate the reduced level of side lobes for the DW‐CRTs, whereas only the EPSI acquisition resulted in a shape of the spatial response similar to that of MPRAGE. The suppression of side lobes for DW‐CRTs (52 and 44 dB for *α* = 1 and *α* = 1.71, respectively) was better than that of EPSI (39 dB) and RE‐CRT (26 dB) (Supporting Figure 2). In addition, the expanded *k*‐space coverage used for DW CRT with *α* = 1.71 maintains the desired SRF, whereas DW CRT with *α* = 1 results in a broadened SRF. The aliasing‐free region for DW CRT with *α* = 1.71 is smaller than that of DW CRT with *α* = 1.

**Figure 3:**
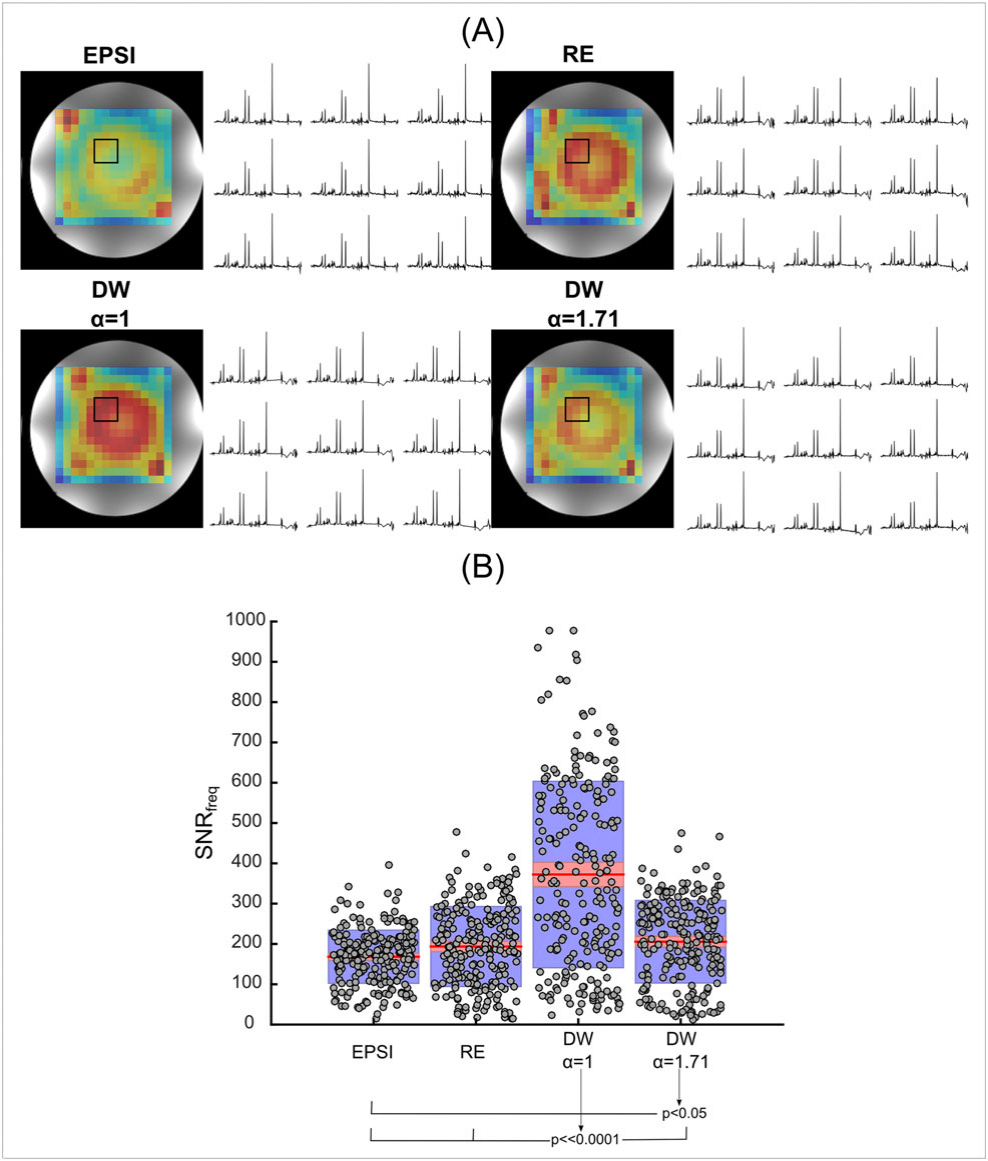
Comparison of spectra acquired from the Braino phantom with EPSI, RE and DW‐CRTs. A, water images with a final grid of 32 × 32 (FOV = 240 mm × 240 mm with a nominal voxel dimension of 0.56 mL) obtained using the first time point of the water FID and spectra belonging to the region inside the black box. B, the statistical comparison of SNR_freq_ from a localized grid of 15 × 15 between different trajectories as points overlaid on a 1.96 × standard error of the mean (SEM) in red and an SD in blue. Post hoc comparisons revealed significant differences in SNR as indicated by the arrowed line connected to the relevant pairs of measurements with horizontal lines. The non‐uniform magnitude of the water images is a result of the *B*_1_ inhomogeneity at 7 T

Figure 3A shows representative EPSI and CRT spectra from the Braino phantom. As illustrated in a 3 × 3 grid marked on the reconstructed water image, metabolite spectra from the DW‐CRTs resulted in a visually discernible improvement in SNR_freq_. The multiple comparison of SNR_freq_ from a localized grid of 15 × 15 voxels between different trajectories is given in Figure 3B (*F*(3, 896) = 99.24, *p* ≪ 0.0001, *η* ^2^ = 0.25). Although the mean SNR of all CRT approaches resulted in higher SNR, the post hoc analysis revealed that only the DW‐CRT with *α* = 1 and *α* = 1.71 improved the SNR significantly compared with the EPSI (*p* ≪ 0.0001 and *p* < 0.03, respectively). The increase in SNR of RE‐CRT versus EPSI (*p* = 0.22) and that of DW‐CRT with *α* = 1.71 versus RE‐CRT was not significant (*p* = 0.80). DW‐CRT with *α* = 1 resulted in the highest SNR compared with the other trajectories (*p* ≪ 0.0001).

### 3.3 *In vivo* comparison of the DW CRTs with EPSI and RE CRT

**Figure 4:**
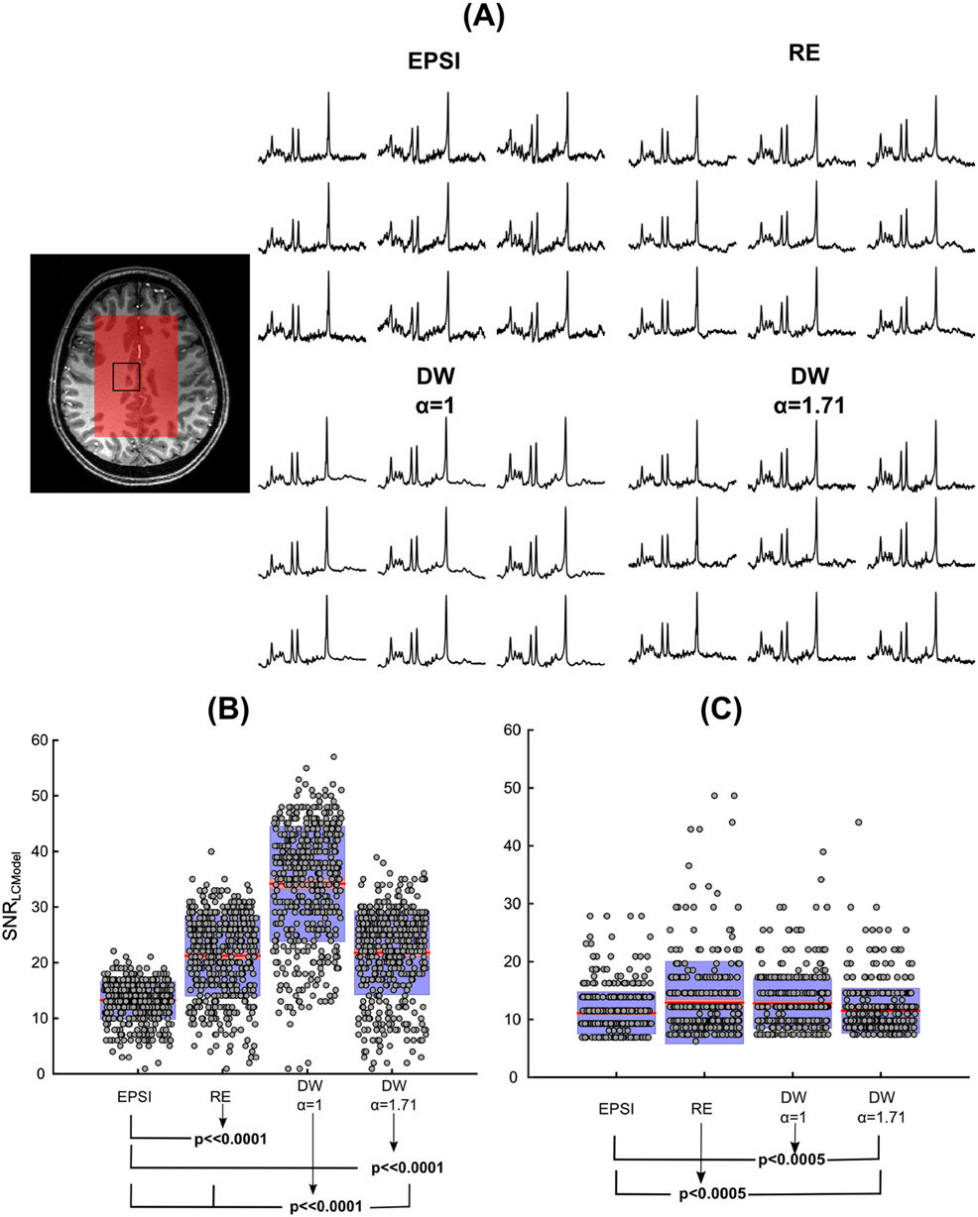
Comparison of *in vivo* spectra acquired with EPSI, RE and DW‐CRTs. A, semi‐LASER localization overlaid on the high‐resolution *T*_1_‐weighted MPRAGE image of the slice and spectra belonging to the region inside the black box. B,C, the statistical comparison of SNR_lcmodel_ and LW_lcmodel_ from a localized grid of 13 × 9 between different trajectories as points overlaid on a 1.96 × SEM in red and an SD in blue. Post hoc comparisons revealed significant differences in SNR_lcmodel_ and LW_lcmodel_ as indicated by arrowed lines connected to the relevant pairs of measurements with horizontal lines

Figure 4 shows the representative EPSI and CRT spectra from a volunteer. As illustrated on a 3 × 3 grid, metabolite spectra from DW‐CRTs resulted in a visually discernible improvement in SNR in the spectra (Figure 4A). The multiple comparisons of SNR_lcmodel_ (*F*(3, 1868) = 616,64, *p* ≪ 0.0001, *η* ^2^ = 0.49) and LW_lcmodel_ (*F*(3, 1868) = 15.41, *p* ≪ 0.0001, *η* ^2^ = 0.02) between different trajectories measured from a grid of 13 × 9 voxels of all volunteers is given in Figure 4B,C. The post hoc analysis revealed that all CRTs improved the SNR significantly when compared with EPSI (*p* ≪ 0.0001). The difference in SNR of DW‐CRT with *α* = 1.71 versus RE was not significant (*p* = 0.68). In agreement with phantom results, the DW‐CRT with *α* = 1 resulted in the highest SNR (*p* ≪ 0.0001). As shown in Figure 4C, post hoc analysis revealed that RE‐CRT and DW‐CRT with *α* = 1 resulted in significantly higher LW_lcmodel_ compared with the EPSI (*p* ≪ 0.0001) and DW *α* = 1.71 trajectories (*p* < 0.0005). The difference in LW_lcmodel_ between DW‐CRT with *α* = 1 and RE was not significant (*p* = 0.98).

**Figure 5:**
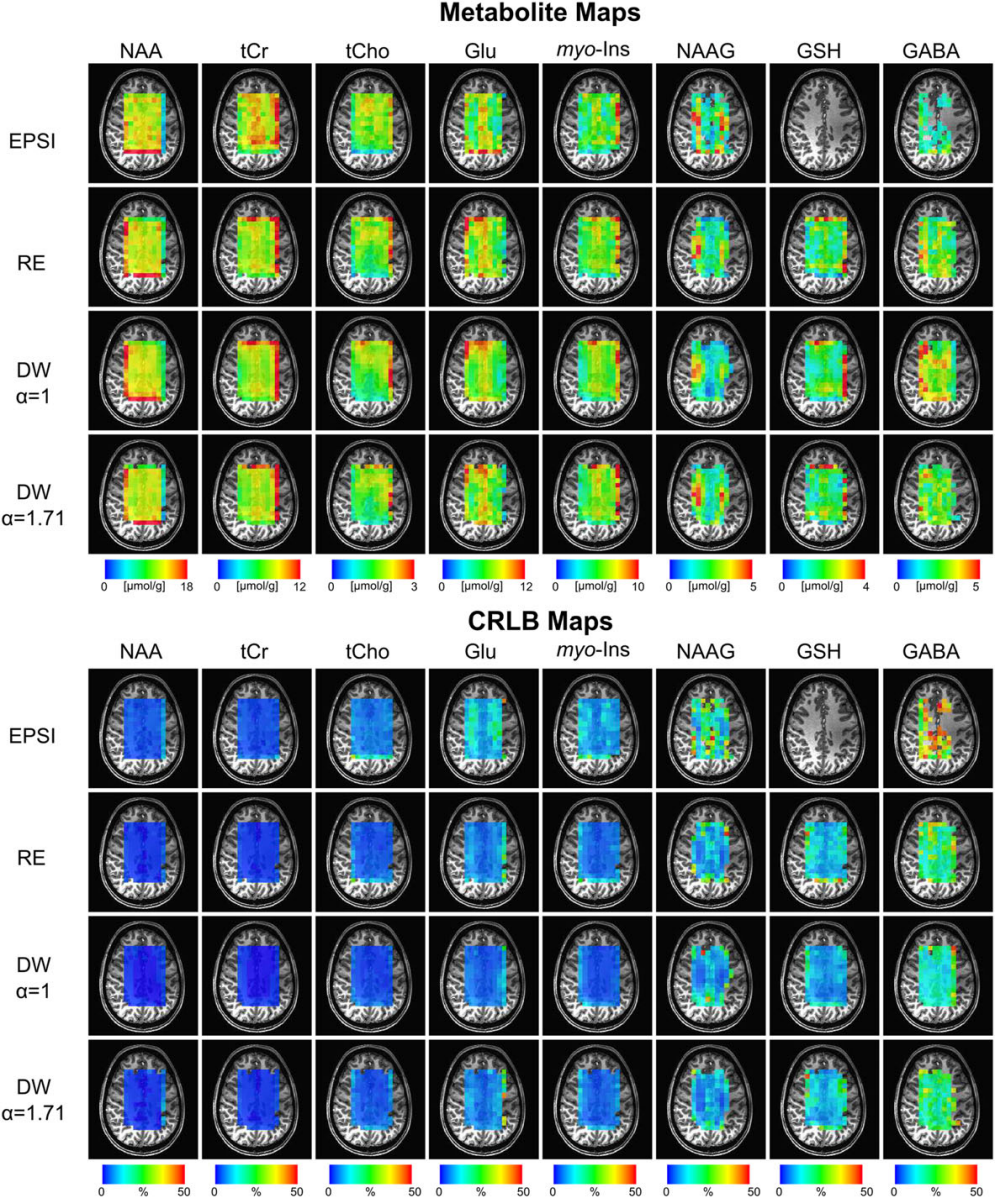
Metabolite and CRLB distribution maps obtained with EPSI, RE and DW‐CRTs from a subject. Absolute metabolite concentration maps from a localized grid of 13 × 9 (a nominal voxel dimension of 0.56 mL) for NAA, tCr, tCho, Glu, *myo*‐ins, NAAG, GSH and GABA overlaid on an anatomical image

Figure 5 shows metabolite and CRLB maps in the brain of a volunteer. A VOI (110 mm × 80 mm × 10 mm, in‐plane resolution 7.5 mm × 7.5 mm) was positioned centrally in the brain by reference to the MPRAGE scan. The CRLB values of most of the metabolites (i.e. NAA, tCr, tCho, GSH, Glu, NAAG, *myo*‐Ins and GABA) were lower than 50% in most of the voxels for all trajectories except for EPSI (Supporting Table 1). The multiple comparisons of CRLB between different trajectories measured from fully excited voxels (13 × 9, 117 voxels) of all volunteers and detected metabolites are given in Supporting Table 1. Due to the lower SNR of EPSI, CRLBs of EPSI were significantly higher compared with CRT trajectories, and LCModel quantification of NAAG, GABA and GSH was severely affected. For instance, metabolites were quantified with a mean CRLB of less than 30% in more than 77% of the fully excited voxels (13 × 9) for all CRTs using a grid of 32 × 32. However, with EPSI GABA and GSH were only quantifiable in 54% and 6.4% of fully excited voxels, with mean CRLBs of 35.9% and 37.2%, respectively.

### 3.4 *In vivo* comparison of the high‐resolution DW CRT (*α* = 1) with RE CRT

**Figure 6:**
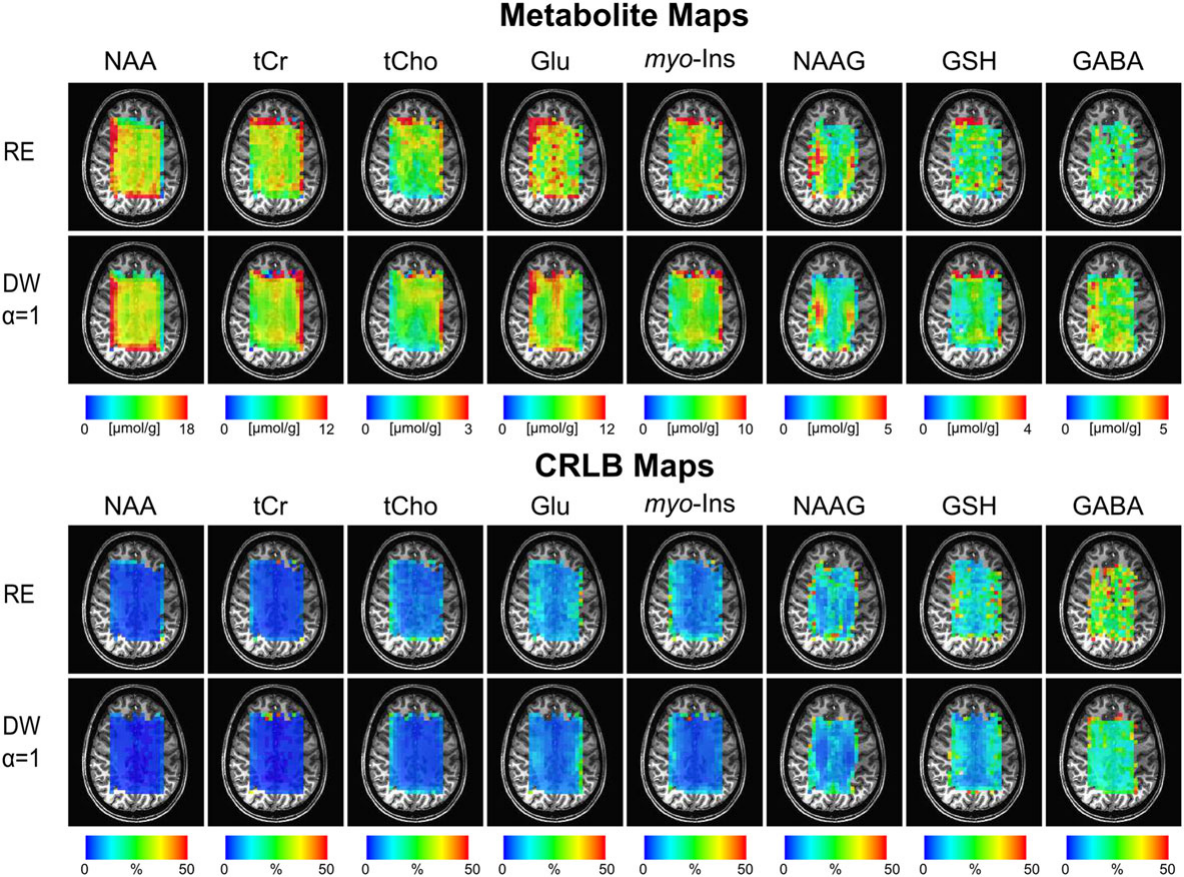
High‐resolution metabolite and CRLB distribution maps obtained with RE and DW‐CRT with *α* = 1 from all subjects. Absolute metabolite concentration maps from a localized grid of 22 × 14 (a nominal voxel dimension of 0.25 mL) for NAA, tCr, tCho, Glu, *myo*‐ins, NAAG, GSH and GABA overlaid on an anatomical image

Figure 6 shows high‐resolution (5 mm × 5 mm) metabolite brain maps from all the volunteers. Due to hardware limitations, high‐resolution measurements could only be conducted for RE‐CRT and DW‐CRT with *α* = 1. CRLB and metabolite maps were complementary to each other for both methods; metabolites with low concentrations showed high CRLBs.

**Figure 7:**
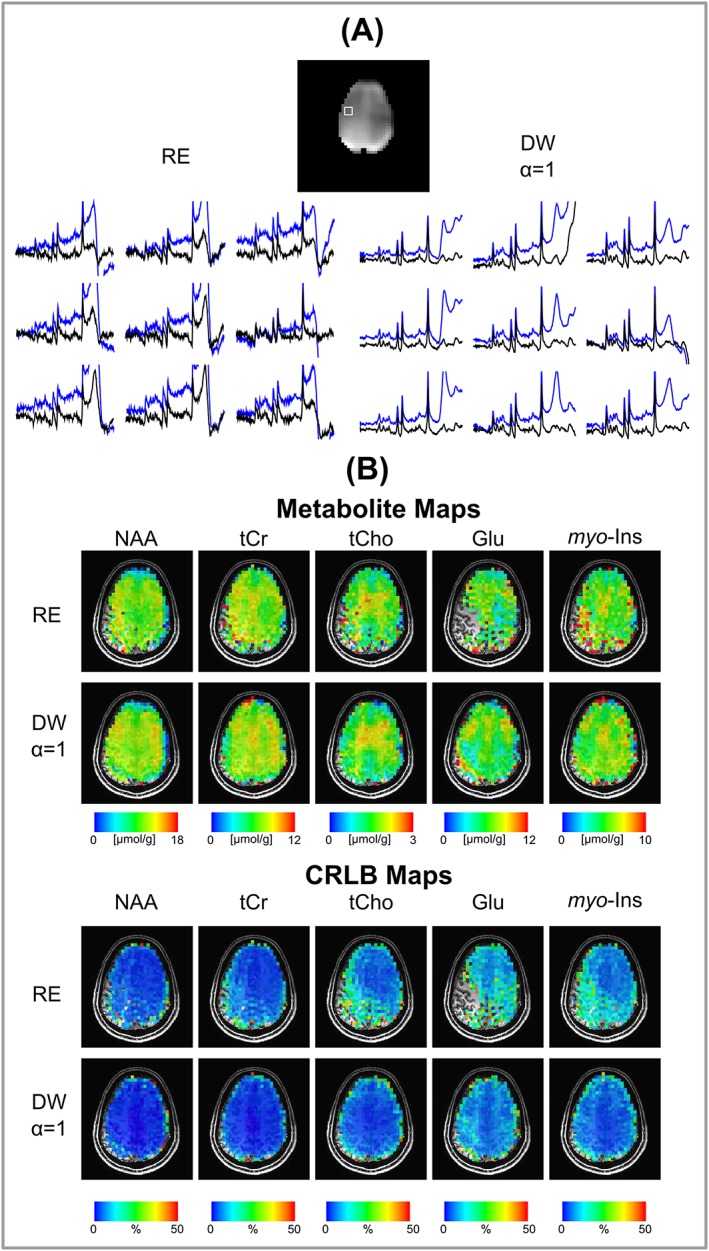
A, nine representative spectra with (black) and without (blue) a lipid‐basis penalty algorithm inside the region of interest marked on the whole brain slice water image (5 × 5 × 10 mm^3^, a nominal voxel dimension of 0.25 mL) of the RE and DW‐CRT with *α* = 1 MRSI acquisitions. B, absolute metabolite and CRLB concentration maps of NAA, tCr, tCho, Glu and *myo*‐ins overlaid on an anatomical image

The feasibility of whole brain slice acquisition was tested with high‐resolution trajectories (0.25 mL using RE‐CRT and DW‐CRT with *α* = 1) in a healthy volunteer. Example spectra marked on the reconstructed water image with a final grid of 48 × 48 are illustrated in Figure 7A, where the spectra before and after L2‐based lipid suppression are compared for both trajectories. Figure 7B shows metabolite and CRLB maps of five important brain metabolites with LCModel for RE‐CRT and DW‐CRT with *α* = 1. Similar metabolite distributions were observed (Supporting Figure 3) when low‐resolution trajectories were used for whole brain slice acquisition (0.56 mL using EPSI, RE and DW‐CRTs).

## 4 DISCUSSION

This study demonstrates that short‐*T*_E_ 2D MRSI data at 7 T can be obtained using DW‐CRTs for fast MRSI acquisition. Two different DW‐CRT sampling schemes (*α* = 1 and *α* = 1.71, corresponding to lower and higher effective spatial resolution respectively) were demonstrated and compared with RE‐CRT and EPSI. Phantom and simulation findings showed that reasonable image quality was preserved with increased SNR using DW‐CRTs. The improved spectral quality offered by DW‐CRTs, as well as the SNR gain of UHF, enabled reliable quantification of low‐concentration metabolites such as NAAG, GSH and GABA from a higher number of voxels compared with RE‐CRT and EPSI. The metabolite concentration values measured using the short‐*T*_E_ MRSI of all trajectories for different tissues were consistent with the literature (see below). Finally, the feasibility of whole brain slice MRSI using DW‐CRT in a healthy volunteer was demonstrated. To our knowledge, this is the first study to have validated the use of DW‐CRT for short‐*T*_E_ MRSI.

Several methods have been proposed to minimize truncation artifacts in MRSI. One common approach is to apply *k*‐space apodization in either post‐processing or data acquisition. Post‐processing with apodization functions, such as a Hanning window, results in a reduction in the magnitude of the side lobes, but the main lobe of the SRF is typically broadened.^37^ However, spatial resolution is preserved and *k*‐space coverage is extended when apodization is used. Furthermore, it has been established that shaping the SRF by manipulating the density of the acquired data incurs no penalty in SNR,^14^ and is better from an SNR perspective than post hoc density weighting or apodization,^32^ which has also been demonstrated in applications such as functional MRI.^38^ For the DW‐CRTs, the sampling density gradually decreased as the trajectory moved away from the origin using a radial Hanning window (Figure 1B). DW‐CRT with *α* = 1 resulted in a broadened SRF, since it is spatially similar to post‐processing with apodization, although without the SNR penalty. However, DW‐CRT with *α* = 1.71 is expected to maintain the desired spatial resolution due to the extended *k*‐space coverage.

For the simulation and phantom measurements, parameters were chosen to match *in vivo* experiments (Table 1). First, in agreement with simulations, the DW‐CRT designs resulted in a reduction in the magnitude of side lobes in the image of SRFs, which is similar to *k*‐space apodization post‐processing used to reduce truncation artifacts.^39^ Although all trajectories had similar FWHMs for SRF experiments (Figure 2B and Supporting Figure 2), EPSI and DW‐CRT with *α* = 1.71 resulted in improved resolution compared with RE‐CRT and DW‐CRT with *α* = 1. On close inspection of the SRFs, it can be seen that the desired SRF shape was only obtained by EPSI and DW‐CRT with *α* = 1.71. In contrast, DW‐CRT with *α* = 1 resulted in a loss of spatial resolution due to a wider SRF. This drawback is clearly illustrated in the images of the resolution phantom experiment (Figure 2A). In addition, given the fact that post hoc weighting was not performed in the reconstruction of the DW‐CRT data, the SRF characteristics resulted in higher SNR after reconstruction. Thus, when DW‐CRT with *α* = 1 was used there was a further increase in SNR due to the wider SRF, although this was also accompanied by the largest spread of SNR values.

It is expected that the noise sensitivity of the interlaced Fourier transform of CRT data is offset by the inverted readout gradient trajectories since the delay between *k*‐space locations is uniform (Supporting Figure 1b), whereas EPSI data are more sensitive to noise since the delay between the odd and even *k*‐space points is non‐uniform and dependent on *k*‐space location and results in noise amplification (Supporting Figure 1a). Thus, SNR differences between EPSI and CRTs originate from not only SRF characteristics, but also the different noise amplification behaviors of the interlaced Fourier transform of CRTs and EPSI trajectories. An alternative approach to alleviate the noise amplification of EPSI would be the use of a slight time shift in temporal interleaves for EPSI. However, this approach would increase the acquisition duration compared with CRTs, since spatial encoding along the readout direction is achieved with the alternating gradient while phase encoding is still used along the other spatial dimension.

Simulation and phantom experiment findings were further supported by *in vivo* experiments. In agreement with phantom experiments, DW‐CRTs resulted in higher SNRs as estimated by LCModel (Figure 4B). Similarly to the phantom measurements, the highest mean SNR_lcmodel_ with highest SNR SD was obtained by using DW‐CRT *α* = 1. Finally, the frequency dimension was sampled with uniform density in all trajectories for each *k*‐space point, and line‐broadening effects in the spectral dimension in DW‐CRTs were not expected. However, increased LW_lcmodel_ estimation of LCModel of RE‐CRT and DW‐CRT with *α* = 1 was observed due to the deterioration from the shape of the desired SRF due to the side lobes and wider SRF, respectively.

For the MRSI acquisition with a resolution of 0.56 mL the achieved *in vivo* spectral quality allowed reliable quantification of major brain metabolites with a CRLB of less than 50% using LCModel analysis. Concentration distributions of metabolites quantified in this study were in good agreement with different trajectories and previously reported values acquired from the same brain locations, and revealed significant variations between different brain tissues (Figure 5). The findings were in agreement with previous MRS studies of those anatomical locations most similar to the regions presented here. For example, Glu, tCr and *myo*‐Ins showed higher concentrations in the region of gray matter (GM) compared with white matter (WM).^6^, ^8^, ^40^ The concentration of NAA had a fairly homogeneous distribution, consistent with previous MRS studies,^6^, ^8^, ^40^ In addition, we found elevated tCho, specifically tCho/tCr, in WM in comparison with GM.^6^, ^8^, ^40^ For low‐concentration metabolites, NAAG, GSH and GABA could not be quantified for EPSI due to the low SNR. In line with previous studies, NAAG was observed with a higher concentration in the WM, whereas GSH was higher in the GM.^8^ However, in contrast to previous literature,^8^, ^41^ we did not observe higher concentration of GABA in GM, which might be due to the use of simulated macromolecules. Finally, as was expected from simulation and phantom findings, DW CRT with *α* = 1 resulted in a smoother metabolite distribution.

In the case of high‐resolution (0.25 mL) MRSI acquisition, experiments were only conducted for DW‐CRT with *α* = 1 and RE‐CRT due to hardware limitations. Concentration distributions of metabolites quantified in this study were in good agreement with previously reported values acquired from the same brain locations. Due to the improved SNR and side lobe reduction, DW‐CRT with *α* = 1 resulted in quantification of metabolites from a higher number of voxels compared with the RE‐CRT approach. Similarly to the low‐resolution findings, DW‐CRT with *α* = 1 resulted in a spatially smoother metabolite distribution. Finally, in agreement with the limited VOI, the high‐quality metabolic mapping of five important brain metabolites taken from the whole brain slice allowed the visualization of metabolites in relation to the underlying anatomical structures.^6^, ^8^

There remain several limitations of the implemented methods. First, the use of broadband adiabatic pulses for semi‐LASER localization resulted in high SAR, which was compensated by the use of a long *T*_R_ of 4 s, with a cost of increased total acquisition duration. The total acquisition duration can be reduced if GOIA pulses^24^ are used instead of selective adiabatic‐full‐passage refocusing pulses, with the potential benefit of reducing total SAR and *T*_R_. Since the DW‐CRTs are not specific to the particular type of localization that was used in this study, using short‐*T*_R_ FID‐MRSI with a reduced readout duration may also reduce the acquisition duration and SAR.^7^, ^42^ Fast *k*‐space trajectories offer reduced acquisition duration for MRSI but suffer from discrepancies between their desired and true trajectories due to system imperfections (such as eddy currents, field oscillations due to gradient vibration, gradient delay and magnetic field drift). Although the CRT, which uses concentrically circular trajectories, is less sensitive to system imperfections compared with other fast acquisition trajectories,^17^ there might be potential to minimize these imperfections if the actual gradient waveforms generated by the scanner were obtained using NMR field probe arrays and used as an input for the reconstruction.^43^ Finally, the exact quantification of peaks might be affected by the use of simulated macromolecules.^44^

In conclusion, we have developed and demonstrated DW‐CRT acquisitions subject to constraints imposed by hardware limitations and the Nyquist and azimuthal sampling criteria. DW‐CRTs with different *α* parameters were used to acquire spectra for high‐resolution *in vivo* MRSI at UHF on healthy volunteers and compared with EPSI and RE‐CRT where the imaging time and voxel size were fixed. The DW‐CRT data demonstrated significantly reduced side lobes together with an increased SNR. As illustrated for the *in vivo* whole brain slice feasibility measurement, the improvements of DW‐CRTs will offer further impact if this technique is translated to clinical 3 T scanners for brain MRSI measurements, since CRT trajectories not only improve the SNR and reduce side lobes, but also offer time efficiency compared with EPSI by sampling *k*‐space in *k_x_* and *k_y_* directions simultaneously.^17^, ^18^

## Supporting information


**Supporting Figure 1** k‐space trajectories of EPSI and concentric rings spectroscopic imaging: (a) a representation of the traversal of the k_x_‐t trajectory achieved during the data acquisition in an EPSI sequence. With the use of odd and even echoes, the spectral bandwidth (SBW) was doubled with a cost of noise amplification since the time interval between odd and even echoes is non‐uniform and dependent on k‐space location^1^. (b) The representation of the traversal of the k_x_k_y_‐t trajectory achieved during the data acquisition in a CRT sequence. With the use of inverted readout gradients, the spectral bandwidth (SBW) was doubled without causing any noise amplification since the time between k‐space of inverted and non‐inverted gradient trajectories, 1/SBW, is constant and independent of the k‐space location.
**Supporting Figure 2** 1D SRF profiles along the x‐axis of all trajectories used for SRF phantom experiments. The y‐axis is on a logarithmic scale to illustrate lipid suppression performance. The lipid suppression performance was calculated in the gray shaded area. The DW‐CRT with α = 1 has significantly lower side lobes, whereas it results in a wider main lobe.
**Supporting Figure 3** Metabolite and CRLB distribution maps obtained with EPSI, RE and DW‐CRTs from a representative subject. Absolute metabolite concentration maps from the whole brain slice acquisition with a nominal voxel dimension of 0.56 mL for NAA, tCr, tCho, Glu, *myo*‐Ins are overlaid on an anatomical image.
**Supporting Table 1** The number of voxels successfully fitted with a CRLB <50% for NAA, tCr, tCho, Glu, *myo*‐Ins, NAAG, GSH and GABA and their mean CRLBs (mean ± SD). The multiple comparisons of CRLB between different trajectories measured from a grid of 13x9 voxels (a nominal voxel dimension of 0.56 mL) of all volunteers are shown in the last column (F value, the effect size).
