# Supplementary material for: Density‐weighted concentric rings k‐space trajectory for 1H magnetic resonance spectroscopic imaging at 7 T
Source: NMR Biomed. 2017 Oct 18;31(1):e3838. doi: 10.1002/nbm.3838 (PMC5969060; doi:10.1002/nbm.3838)
Supplement: Supplementary file 1 — Supporting Figure 1 k‐space trajectories of EPSI and concentric rings spectroscopic imaging: (a) a representation of the traversal of the kx‐t trajectory achieved during the data acquisition in an EPSI sequence. With the use of odd and even echoes, the spectral bandwidth (SBW) was doubled with a cost of noise amplification since the time interval between odd and even echoes is non‐uniform and dependent on k‐space location1. (b) The representation of the traversal of the kxky‐t trajectory achieved during the data acquisition in a CRT sequence. With the use of inverted readout gradients, the spectral bandwidth (SBW) was doubled without causing any noise amplification since the time between k‐space of inverted and non‐inverted gradient trajectories, 1/SBW, is constant and independent of the k‐space location. Supporting Figure 2 1D SRF profiles along the x‐axis of all trajectories used for SRF phantom experiments. The y‐axis is on a logarithmic scale to illustrate lipid suppression performance. The lipid suppression performance was calculated in the gray shaded area. The DW‐CRT with α = 1 has significantly lower side lobes, whereas it results in a wider main lobe. Supporting Figure 3 Metabolite and CRLB distribution maps obtained with EPSI, RE and DW‐CRTs from a representative subject. Absolute metabolite concentration maps from the whole brain slice acquisition with a nominal voxel dimension of 0.56 mL for NAA, tCr, tCho, Glu, myo‐Ins are overlaid on an anatomical image. Supporting Table 1 The number of voxels successfully fitted with a CRLB <50% for NAA, tCr, tCho, Glu, myo‐Ins, NAAG, GSH and GABA and their mean CRLBs (mean ± SD). The multiple comparisons of CRLB between different trajectories measured from a grid of 13x9 voxels (a nominal voxel dimension of 0.56 mL) of all volunteers are shown in the last column (F value, the effect size). [file NBM-31-na-s001.docx]

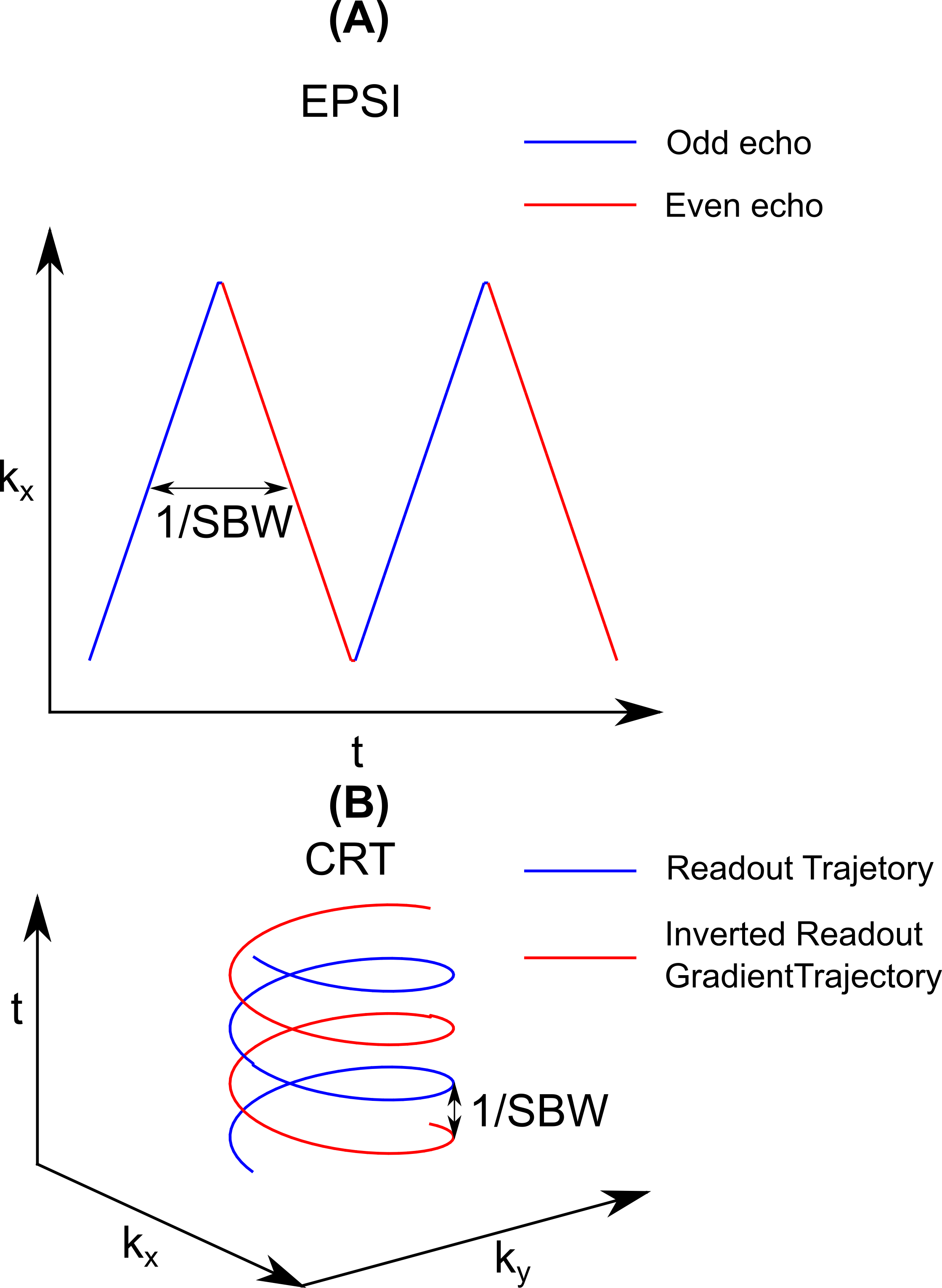


**Supporting Figure 1** k-space trajectories of EPSI and concentric rings spectroscopic imaging: (a) a representation of the traversal of the k_x_-t trajectory achieved during the data acquisition in an EPSI sequence. With the use of odd and even echoes, the spectral bandwidth (SBW) was doubled with a cost of noise amplification since the time interval between odd and even echoes is non-uniform and dependent on k-space location^1^. (b) The representation of the traversal of the k_x_k_y_-t trajectory achieved during the data acquisition in a CRT sequence. With the use of inverted readout gradients, the spectral bandwidth (SBW) was doubled without causing any noise amplification since the time between k-space of inverted and non-inverted gradient trajectories, 1/SBW, is constant and independent of the k-space location.


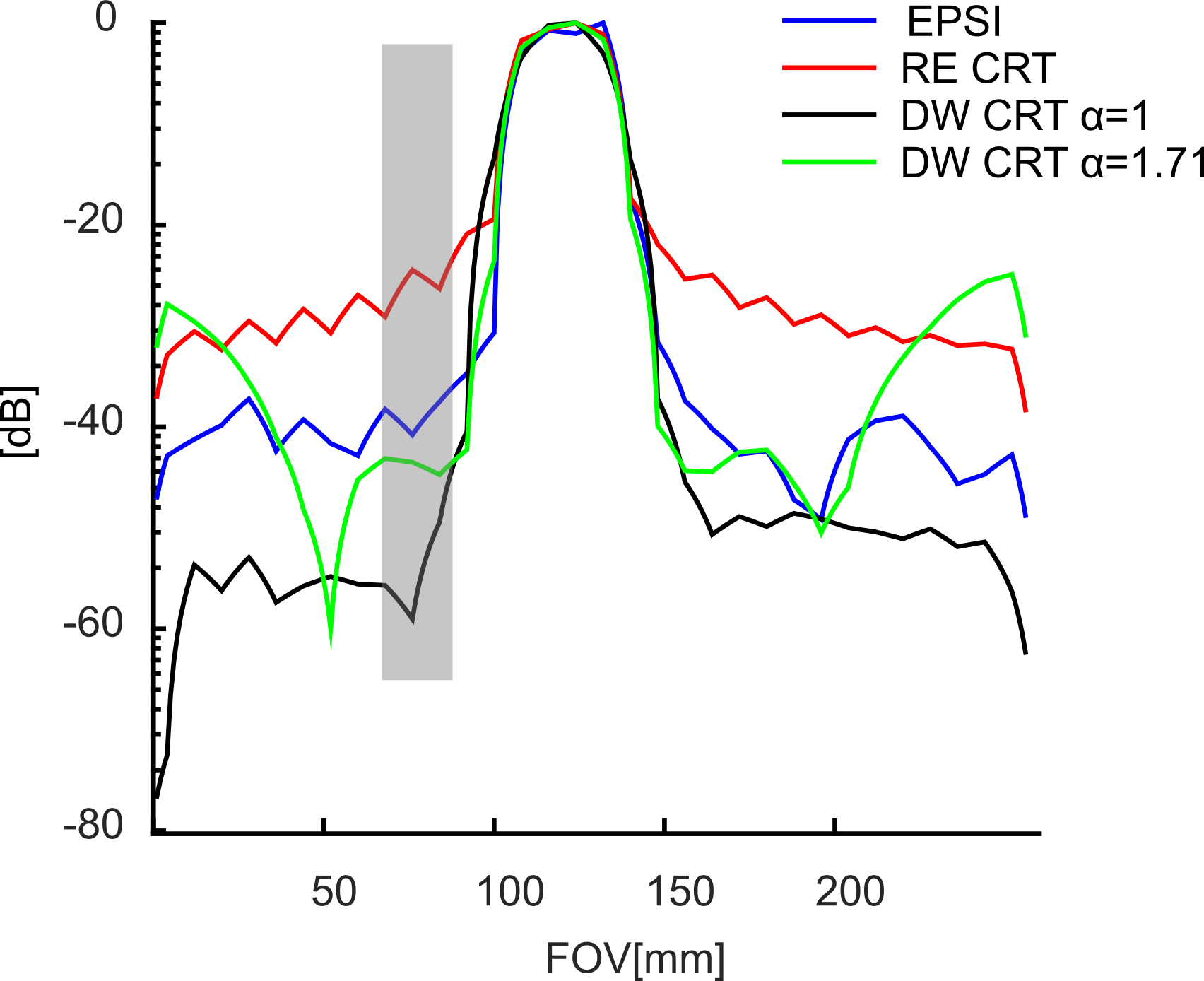


**Supporting Figure 2** 1D SRF profiles along the x-axis of all trajectories used for SRF phantom experiments. The y-axis is on a logarithmic scale to illustrate lipid suppression performance. The lipid suppression performance was calculated in the gray shaded area. The DW-CRT with α=1 has significantly lower side lobes, whereas it results in a wider main lobe.


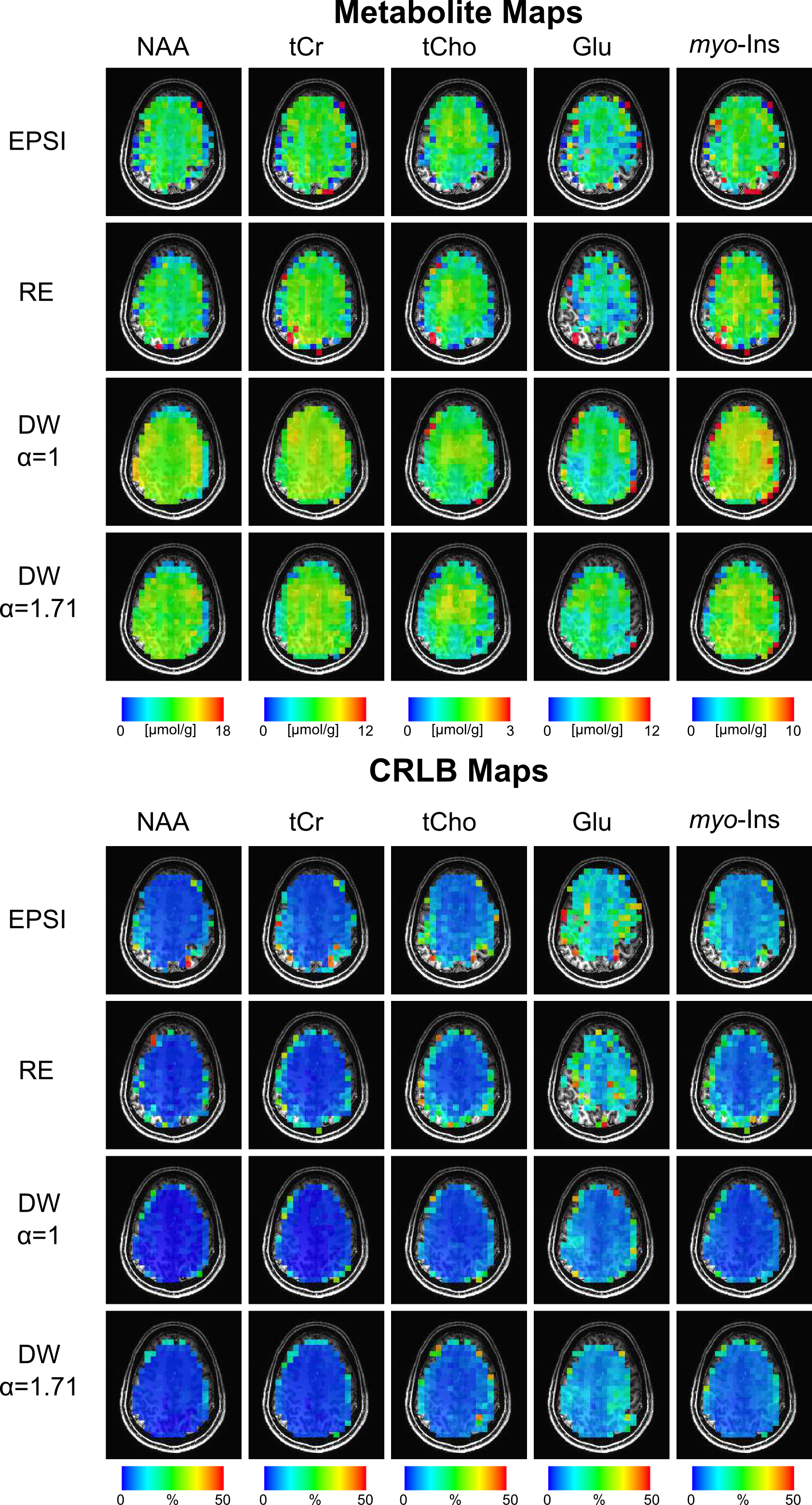


**Supporting Figure 3**

Metabolite and CRLB distribution maps obtained with EPSI, RE and DW-CRTs from a representative subject. Absolute metabolite concentration maps from the whole brain slice acquisition with a nominal voxel dimension of 0.56 mL for NAA, tCr, tCho, Glu, *myo*-Ins are overlaid on an anatomical image.

**Supporting Table 1** The number of voxels successfully fitted with a CRLB <50% for NAA, tCr, tCho, Glu, *myo*‐Ins, NAAG, GSH and GABA and their mean CRLBs (mean ± SD). The multiple comparisons of CRLB between different trajectories measured from a grid of 13x9 voxels (a nominal voxel dimension of 0.56 mL) of all volunteers are shown in the last column (F value, the effect size).

|  | **EPSI** | | **RE-CRT** | | **DW-CRT α=1.0** | | **DW-CRT α=1.71** | | **ANOVA** |
| --- | --- | --- | --- | --- | --- | --- | --- | --- | --- |
| Metabolites | CRLB  (%) | Number of Voxels | CRLB  (%) | Number of Voxels | CRLB  (%) | Number of Voxels | CRLB  (%) | Number of Voxels | F value, the effect size |
| **NAA** | 3.97±1.73 | 115.75±1.5 | 2.41±1.27 | 114±0.57 | 1.56±0.79 | 116.25±0.5/117 | 2.33±1.38 | 113.25±2.06 | F(3,1835)=264.82, 0.30 |
| **tCr** | 3.37±1.50 | 116.5±0.57 | 2.63±1.94 | 114.75±0.5 | 1.62±0.84 | 116.25±0.5 | 2.47±2.22 | 113.5±2.08 | F(3,1840)=82.83, 0.13 |
| **tCho** | 6.38±3.81 | 116.25±0.5 | 5.13±4.63 | 114.2±-0.5 | 3.31±2.28 | 116.25±-0.5 | 4.75±2.83 | 113.25±2.21 | F(3,1836)=60.40, 0.08 |
| **Glu** | 11.50±5.93 | 108±2.45 | 7.74±4.40 | 114.5±0.57 | 5.34±2.99 | 116.25±0.5 | 7.75±5.61 | 110.50±2.08 | F(3,1793)=122.63,0.17 |
| **myo-Ins** | 8.52±4.06 | 115.5±0.57 | 6.40±4.35 | 114.2±-0.95 | 4.13±2.12 | 116.25±0.5 | 5.92±3.91 | 113.5±2.0817 | F(3,1834)=109.43,0.15 |
| **NAAG** | 18.68±10.72 | 90.5±7.85 | 13.40±9.09 | 90.5±6.19 | 12.66±8.89 | 94.25±4.5 | 11.56±7.74 | 96.0±7.78 | F(3,1481)=43.78,0.08 |
| **GSH** | 37.20±9.56 | 7.5±3 | 13.84±8.00 | 108.25±3.86 | 9.29±4.96 | 114.75±0.5 | 13.58±7.56 | 106.5±1.0 | F(3,1344)=164.51,0.26 |
| **GABA** | 35.99±7.86 | 63.25±11.35 | 22.85±7.88 | 95.7±-3.2 | 16.84±6.74 | 106.25±4.78 | 22.81±7.82 | 94.25±1.5 | F(3,1434)=343.26,0.41 |

1. Metzger G, Hu X. Application of interlaced Fourier transform to echo-planar spectroscopic imaging. *J Magn Reson.* 1997;125(1):166-170.
